# Wood Rendered Incombustible

**Published:** 1877-05

**Authors:** 


### WOOD RENDERED INCOMBUSTIBLE.

It has been demonstrated by the most rigid experiments that wood immersed in a "pickle" of a solution of tungstate of soda cannot be ignited under any of the ordinary conditions to which it may be exposed. The tungstate is made by the addition of tungstate of lime to hydrochloric acid and salt, affording as a by-product chloride of lime in large quanities. The action of the tungstate, upon soft woods is to render them quite hard as well as incombustible, and it also acts as a preventive against dry rot.

Sticks and boards of the prepared wood have been saturated with kerosene oil and set on fire; the oil burned off entirely without igniting the wood. Two small houses have been built, one of ordinary pine wood, the other of the prepared wood, and fires of great urgency kindle in each. The one of ordinary wood was quickly consumed, while the other was left only slightly charred.

There is no doubt that all the wood-work of theatres can be made practically incombustible, and at no very great cost. A bath of the tungstate of soda, consisting of several thousand gallons, can be employed, and sufficient wood to construct all the scenery and appliances of a theatre can be prepared in a few weeks' time, and the painted canvas also can be treated in the same way. Even if oil colors were used on the canvas, we think it could not be readily ignited.

The attention of theatre managers should be called to this subject, and the authorities of cities should insist upon having every means used which modern science suggests to prevent fires in public buildings.—*Boston Journal of Chemistry.*

